# Considering Cause and Effect of Immune Cell Aging on Cardiac Repair after Myocardial Infarction

**DOI:** 10.3390/cells9081894

**Published:** 2020-08-13

**Authors:** Stephanie W. Tobin, Faisal J. Alibhai, Richard D. Weisel, Ren-Ke Li

**Affiliations:** 1 Division of Cardiovascular Surgery, Toronto General Hospital Research Institute, University Health Network, Toronto, ON M5T 1P5, Canada; stephanie.tobin@uhnresearch.ca (S.W.T.); Faisal.Alibhai@uhnresearch.ca (F.J.A.); richard.weisel@uhn.ca (R.D.W.); 2 Division of Cardiac Surgery, Peter Munk Cardiac Centre, Toronto General Hospital and University of Toronto, Toronto, ON M5G 2N2, Canada

**Keywords:** aging, inflammation, myocardial infarction, therapeutics, immune system

## Abstract

The importance of the immune system for cardiac repair following myocardial infarction is undeniable; however, the complex nature of immune cell behavior has limited the ability to develop effective therapeutics. This limitation highlights the need for a better understanding of the function of each immune cell population during the inflammatory and resolution phases of cardiac repair. The development of reliable therapies is further complicated by aging, which is associated with a decline in cell and organ function and the onset of cardiovascular and immunological diseases. Aging of the immune system has important consequences on heart function as both chronic cardiac inflammation and an impaired immune response to cardiac injury are observed in older individuals. Several studies have suggested that rejuvenating the aged immune system may be a valid therapeutic candidate to prevent or treat heart disease. Here, we review the basic patterns of immune cell behavior after myocardial infarction and discuss the autonomous and nonautonomous manners of hematopoietic stem cell and immune cell aging. Lastly, we identify prospective therapies that may rejuvenate the aged immune system to improve heart function such as anti-inflammatory and senolytic therapies, bone marrow transplant, niche remodeling and regulation of immune cell differentiation.

## 1. Introduction

After myocardial infarction (MI), cardiomyocyte death activates cellular and structural remodeling of the heart: Remaining cardiomyocytes take on an additional contractile load, leading to cardiomyocyte hypertrophy, while fibroblasts proliferate, produce extracellular matrices, to help replace lost cardiomyocytes with collagenous scar. The extent of the infarct necrosis is influenced by the collateral circulation which may preserve injured but viable myocardium. Perhaps the most diverse and complex reaction, however, is the immune response which has been shown to influence multiple repair processes. There is increasing interest in developing pharmacological and cell-based therapies that target the immune response to improve cardiac repair following ischemic injury. Anti-inflammatory medications, such as glucocorticoids or non-steroidal anti-inflammatory drugs (NSAIDs) which broadly target inflammation, revealed that these drugs contribute to adverse cardiac outcomes, demonstrating the complex nature of the immune system and the recovery of cardiac function [1,2,3]. More precise control over different populations of immune cells is required to achieve a safe and effective treatment. Another facet to the complexity of putative therapies is aging which, at the cellular level, is inherently associated with reduced cellular functionality and at the tissue level, is associated with both immunological and cardiovascular diseases. This review examines the effect of aging on the immune system and evaluates regenerative immunotherapies that may be tailored to treat cardiovascular pathologies after ischemic injury.

## 2. Typical Immune Responses after Myocardial Infarction

Immune cells are divided into lymphoid and myeloid cell types based on two common progenitor cells. Lymphoid cells include natural killer (NK) cells, T cells and B cells while myeloid cells include basophils, neutrophils, eosinophils and monocytes (the precursor to dendritic cells and bone-marrow-derived macrophages), mast cells and megakaryocytes, which produce anuclear platelets via thrombopoiesis. All immune cells, except for T and B cells, are part of the innate immune response which is designed to (1) recognize damage or pathogen-associated molecular patterns (DAMPs or PAMPs, respectively), (2) amplify the innate immune response, (3) clear pathogens and debris via phagocytosis and (4) activate the adaptive immune response (T and B cells) [4]. Phagocytic cells are a diverse population which clear debris and modulate the microenvironment via cytokine and growth factor release. Phagocytic cells include neutrophils, dendritic cells and macrophages, the latter of which have become an active area of research in cardiac disease because of their diverse role in tissue repair.

The dynamic role of the immune system in cardiac homeostasis and disease has been reviewed extensively [5,6]. Briefly, in the healthy adult heart, resident immune cells include B and T cells, mast cells and macrophages. Using single cell suspensions and flow cytometry, Pinto et al. determined that 9% of non-myocyte cells are leukocytes. This number further breaks down into 7% myeloid cells, which were mostly macrophages, and roughly 1% lymphocytes [7]. Resident cardiac macrophages have an important role in steady-state homeostasis but also regulate cardiomyocyte contraction via electrical coupling [8].

### 2.1. Myeloid Cell Activity after Myocardial Infarction

**Figure 1:**
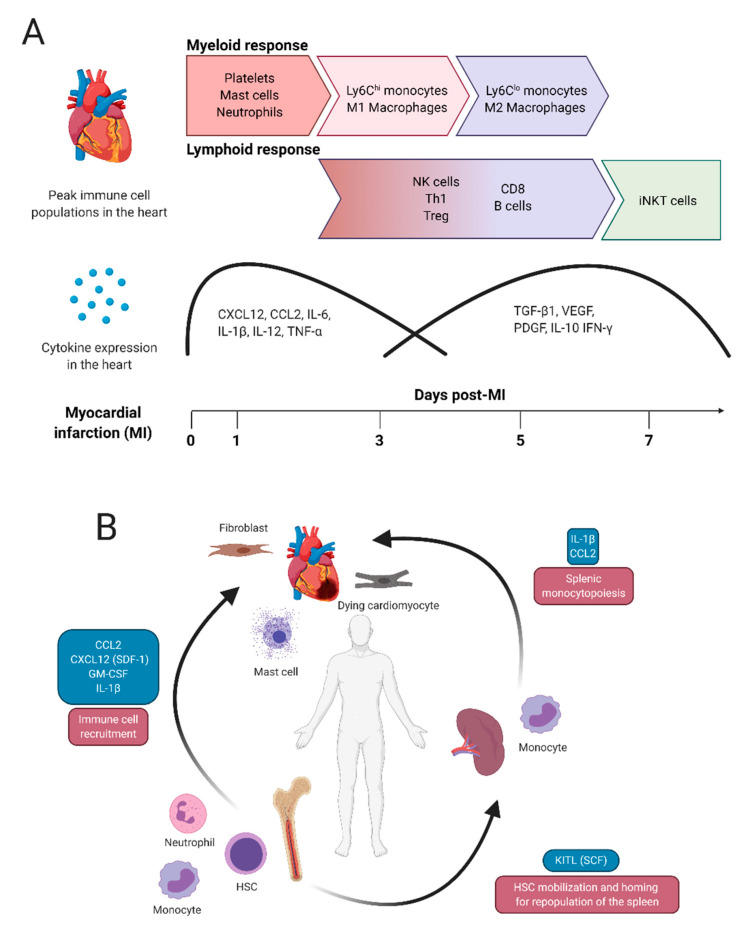
Typical immune cell and tissue responses in the heart after permanent coronary artery occlusion. (**A**) The generalized pattern of immune cell infiltration to the heart after ischemic injury. Here, immune cell infiltrate is subdivided into myeloid and lymphoid populations. Myeloid cell infiltration spans approximately 1–3 days post-MI, while lymphoid cell populations infiltrate the heart at approximately 3–7 days post-MI. (**B**) Systemic tissue response to MI. After MI, signals from the heart (e.g., from degranulating mast cells, dying cardiomyocytes or activated fibroblasts) act on the spleen and bone marrow to initiate tissue repair. The activation of HSCs and migration of immune cells is triggered by a diverse array of molecules, including but not limited to CCL2, CXCL12 (SDF-1), GM-CSF and IL-1β. Monocytopoiesis is regulated by IL-1β and CCL-2. Gradually, HSCs in the bone marrow will repopulate the spleen in part through chemotactic molecules such as KITL (SCF).

The most common cause of acute myocardial ischemia is coronary artery obstruction by rupture of an atherosclerotic plaque and thrombosis of the vessel [9]. When the plaque ruptures, platelets migrate to the disrupted endothelium and adhere to constituents of the vessel wall to form a clot, occluding the vessel, which consequently causes cardiac ischemia [10]. After acute myocardial injury, cardiac-resident, splenic and bone-marrow-derived immune cells participate in two cellular phases, inflammation and resolution, both of which are critical for effective repair. In experimental rodent models of permanent coronary artery occlusion, the cumulative inflammatory phase of all leukocytes typically peaks at 3 days post-infarction which may correspond to 1 to 2 weeks in humans [11]. Unfortunately, less is known about the temporal immune response in humans after myocardial infarction. Within this timeframe are waves of immune cell infiltration, which vary by cell type and cytokine and chemokine production. Figure 1A depicts the general pattern of immune cell and cytokine density within the rodent heart at 1, 3, 5 and 7 days after permanent occlusion. Within the first 24 h, and up to 3 days post-injury, myeloid cells contribute to tissue inflammation while at approximately 5–7 days post-injury, myeloid cell behavior takes on a reparative phenotype and lymphoid cell infiltration increases. Regulating the early innate immune response is important as it can limit infarct size [12]. Rodent models that use ischemia-reperfusion injury have a faster inflammatory response, particularly with respect to the lymphoid population [13].

After MI, the cardiac microenvironment consists of ischemic and dead cardiomyocytes which release DAMPs including cytokines and chemokines such as monocyte chemoattractant protein-1 (MCP-1/CCL2), Interleukin (IL)-1α, IL-6, C-X-C Motif Chemokine Ligand 16 (CXCL16), the macrophage-inhibitory factor (MIF) and CXCL12, which recruit leukocytes to the heart [14]. These molecules act in an autocrine, paracrine and endocrine signaling cascades to amplify pro-inflammatory signals and promote immune cell recruitment (Figure 1B). Resident fibroblasts also contribute to inflammation by releasing granulocyte/macrophage colony-stimulating factor (GM-CSF), which stimulates local leukocyte activity and acts at a distance in the bone marrow, triggering hematopoiesis [15]. Concomitant with the upregulation of HSC chemotactic factors in the infarcted heart, stem cell retention factors such as CXCXL12, are downregulated in the bone marrow due to activation of β3 adrenergic signaling [16]. CCL2 also stimulates proliferation of a subset of primitive HSCs (CD150^+^ CD48^−^ Lineage^−^ Sca-1^+^ c-Kit^+^) which express C-C chemokine receptor 2 (CCR2), thereby contributing to the potent immune reaction that develops post-MI [17]. The release of HSCs from the bone marrow requires matrix metalloproteinase (MMP)-9 activity and if MMPs are inhibited, HSC recruitment is impaired and cardiac function deteriorates [18]. HSC activation and recruitment is an important determinant of cardiac functional recovery after MI as dysfunctional HSCs results in severe, terminal heart failure [18,19].

Platelets are recruited to the ischemic myocardium during the early inflammatory phase in animal models of both permanent coronary occlusion and ischemia-reperfusion (I/R), though permanent occlusion leads to persistent platelet recruitment, spanning 6 to 72 h post-MI [20], while I/R leads to transient platelet infiltration approximately 2 h post-injury [21]. Here, platelets release various pro-inflammatory biomolecules and interact with other leukocytes [22].

Granulocytes also have an important role during the early inflammatory phase. This cell population includes neutrophils, eosinophils, basophils and mast cells which carry a diverse assortment of bioactive molecules such as histamines, proteases and cytokines. Mast cells migrate and mature in resident tissues while all other granulocytes are recruited from the bone marrow in response to pro-inflammatory stimulation. Basophils have a role in autoimmune reactions and can regulate fibrosis, T cell activity and eosinophil recruitment [23,24,25] but have not been shown to have a substantial role in heart repair. Eosinophils are an understudied immune cell population in the ischemic heart. It was recently shown that eosinophil depletion worsened cardiac outcomes after permanent coronary artery occlusion, indicating this immune cell population warrants further investigation [26].

Approximately 8–24 h post-MI (in mice), resident mast cells degranulate, releasing cytokines, such as Tumor Necrosis Factor (TNF)-α, to amplify the inflammatory response, contribute to the death of injured cardiomyocytes and also facilitate immune cell recruitment from the bone marrow and spleen [27]. Mast cells also release proteases that degrade bio-protective molecules such as Insulin-like growth factor (IGF)-1 [28,29]. While mast cells are generally associated with adverse cardiac events, they also contribute to cardiac remodeling as they can promote either fibrosis or collagen degradation via MMP activation, depending on the stimulus [30]. Neutrophils, which have dual roles as phagocytes and granulocytes, are recruited within the first 24 h post-MI. Neutrophils amplify tissue injury via monocyte recruitment and the production of cytokines such as IL-1β, TNF-α, CCL3, and IL-12 [31,32] but later act as an anti-inflammatory switch in macrophages either by modulating the local microenvironment, or by programming macrophage behavior from within after efferocytosis [33]. Conversely, Horckmans et al. suggested that abrogation of all neutrophils has been shown to be detrimental and lead to adverse cardiac remodeling, including uncontrolled fibrosis and reduced macrophage efferocytosis [34].

Monocyte infiltration occurs between 1 and 5 days post-MI and contributes to both inflammatory and reparative phases. Splenic monocytes supply the initial infiltration of monocytes shortly after MI [35,36] in an IL-1β dependent manner [37,38]. As circulating monocytes survive between 1 and 7 days [39], the body also stimulates monocyte differentiation and recruitment from the bone marrow [36]. Progenitors from the bone marrow will later replenish the spleen in a c-Kit dependent manner [16]. In addition to IL-1β, Ly6C^hi^ monocytes are recruited to the heart via MCP-1/CCL2 which acts on CCR2 [40,41,42]. The systemic interaction between heart, spleen and bone marrow during the early phase of cardiac repair after MI is depicted in Figure 1B. Blocking monocyte recruitment to the infarcted heart via CCR2 chemokine receptor antagonist limits inflammation, enhances angiogenesis and maintains beneficial resident cardiac macrophages [43]. Ly6C^lo^ monocytes do not express CCR2 but instead express CX_3_CR1, providing a useful method to sort these two monocyte populations [44]. The first monocyte population to appear is pro-inflammatory Ly6C^hi^ monocytes which differentiate into CCR2^+^ macrophages. As healing proceeds, Ly6C^hi^ monocytes gives rise to Ly6C^lo^ monocytes which differentiate into macrophages at a lower rate and function primarily as pro-reparative cells and survey endothelial cell health for clearance [45,46,47]. The conversion and survival of Ly6C^lo^ monocytes is controlled by Nuclear receptor subfamily 4 group A member 1 (Nr4a1, aka Nur77) [48,49]. While for some time, the ontogeny of bone marrow or splenic-derived cardiac macrophages from Ly6C^hi/lo^ monocytes was unclear, analysis using bone marrow transplant from Nr4a1 knockout mice (*Nr4a1^−/−^*), which lack LyC6^lo^ monocytes, to WT mice showed that both inflammatory and reparative macrophages arise exclusively from Ly6C^hi^ monocytes [49]. Bone marrow replacement with *Nr4a1^−/−^* HSCs resulted in an amplified inflammatory response and fewer reparative macrophages after MI [49].

Macrophages are one of the better characterized immune cells in cardiac disease because of their important functional role in tissue repair. During development, cardiac macrophages develop from the yolk sac and are present in the heart as CCR2^−^ macrophages which promote heart function via cardiomyocyte proliferation and angiogenesis [49,50]. A number of CCR2^−^ tissue-resident cardiac macrophages die after MI, which are then replaced by CCR2^+^ macrophages derived from circulating Ly6C^hi^ monocytes [50]. Resident and bone-marrow-derived macrophages can be identified by using a combination of CCR2, MHC-II, Ly6C, CX_3_CR1, TIMD4, LYVE1 but cell sorting depth and preference varies across studies [43,51,52]. The recent identification of new populations of cardiac macrophages has widened the breadth of macrophage function [43,51,52,53] but for some time, macrophages were grouped into two functional roles after acute injury such as myocardial infarction: M1 (pro-inflammatory) followed by M2 (anti-inflammatory) macrophages. Under this lens, M1 and M2 macrophages are present in sequential functional waves. Initially, M1 macrophages contribute to further tissue damage and cellular digestion to facilitate wound clearance via production of cytokines [54]. Approximately 5–7 days after permanent coronary artery occlusion or I/R, the resolution phase begins and the macrophage population is predominantly M2 [13]. The M2 macrophage response is slightly more complex than classically activated M1 macrophages as three subsets of M2 macrophages have been characterized, depending on the in vitro differentiation conditions. Both M2a and M2c are associated with tissue repair and extracellular matrix deposition, while M2b has an immunomodulatory role [55]. One of the better characterized M2a cytokines is IL-10, which acts on endothelial cells and fibroblasts to promote angiogenesis and deposition of extracellular matrix [56], respectively, and also behaves as an anti-inflammatory. M2 macrophages also secrete factors such as the Transforming Growth Factor (TGF)-β superfamily (e.g., TGF-β1 and Growth differentiation factor (GDF)-15, Vascular endothelial growth factor (VEGF), and Platelet-derived growth factor (PDGF). M2 macrophages also express arginase (ARG) 1 and 2 which facilitate collagen production. Using the permanent occlusion model, M1 TNF-α^+^ macrophages and M2 ARG1^+^ macrophages were quantified at 2-, 5- and 10- days post-MI. TNF-α^+^ macrophages began to decline by day 5 while ARG1^+^ macrophages were still increasing at day 10, corresponding to the functional change in macrophage behavior from pro-inflammatory to reparative [54].

Inflammatory M1 and reparative M2 macrophages are a loose delineation of macrophages based on function. Single cell RNA-seq has revealed that there are at least seven different cardiac macrophage populations in the infarcted heart [41,42], a far cry from the M1 and M2 macrophage dichotomy. Deletion of one macrophage subset, interferon inducible macrophages (IFNICs) was able to improve heart function after MI, demonstrating the therapeutic potential that targeting select groups of cardiac macrophages could have on heart disease [53], though timing will be critical. King et al. demonstrated that limiting the activity of IFNICs via pharmacological inhibitors in mice benefits heart function if administered during the early phase of MI, within the first 48 h [53].

### 2.2. Lymphoid Cell Activity after Myocardial Infarction

Lymphoid cells of the adaptive immune system include B (discovered in the bursa of Fabricius, a lymphoid organ in birds) and T (Thymus) cells which arise from a common lymphoid progenitor (CLP) but mature in the bone marrow or thymus, respectively. Natural killer (NK) cells, which are distinguished by the cell surface marker CD56, also arise from a CLP. NK cells participate in the innate immune response and have a protective role in limiting inflammation in the setting of myocarditis [57]. Their role after myocardial infarction is more complex as they demonstrate pro- and anti-inflammatory potential; however, their infiltration peaks approximately 5 days after permanent occlusion which may indicate a more important role in the latter [13,58,59].

T cells recognize peptides presented by antigen presenting cells such as macrophages, dendritic cells and B cells to mount an appropriate immune response by scaling the reaction up or down. Depending on the antigen present, naïve T cells can be stimulated to form CD4^+^ T helper cells or cytotoxic CD8^+^ T cells. CD4^+^ T helper cells further specialize as they differentiate into effector T cells called Th1, Th2, Th17 and T regulatory (Treg) cells. Treg cells are often grouped as their own T cell subtype because of their role in immuno-suppression [60]. Additional T cells include γδ T cells, Th9, Th22, T follicular helper (Tfh) cells and T follicular regulatory (Tfr) cells but are omitted for the purpose of this review.

The adaptive immune response in the ischemic heart runs the gamut of T cells, B cells and NK cells of varying densities, and arrive approximately 3–7 days post-MI [13]. Their delayed recruitment during the resolution phase correlates with their functional ability to modulate cardiac fibrosis, though some studies demonstrate CD3^+^T cells peaking 1 day post-MI [59]. Thus, the timely recruitment of lymphoid cell recruitment is not as well mapped out as myeloid cells (Figure 1A). From the CD4^+^ cell cohort, Th1 and Treg cells are the most abundant [13]. After acute cardiac injury, Th1 cells migrate to the heart and produce pro-inflammatory cytokines such as Interferon (IFN)-γ, while Th2 and Th17 T cell infiltration is comparatively negligible [13,61]. Th1 recruitment coincides with the immuno-suppressive Treg cell activity. The presence of both pro- and anti-inflammatory CD4^+^ cells may be related to the MI model (permanent occlusion vs. I/R) which demonstrate conflicting roles of CD4^+^ cells on heart repair. Using I/R, Yang et al. demonstrated that antibody depletion of CD4^+^ but not CD8^+^ T cells reduced infarct size [62], indicating a harmful role for CD4^+^ cells in heart repair. In contrast, permanent coronary artery occlusion of CD4^+^ knockout mice increased inflammation, mortality and cardiac rupture, indicating an important role for these cells in wound healing [61]. Further studies with more stringent cellular analyses will be necessary to delineate the different roles of the CD4^+^ T cells. Treg cells have an important role in macrophage polarization as genetic deletion of Treg cells using Foxp3 diphtheria toxin receptor (DTR) mice or anti-CD25 monoclonal antibody-mediated depletion worsened cardiac repair and enhanced M1 macrophage polarization while Treg activation induced M2 polarization and improved wound healing [63]. The recruitment of Treg cells is in line with the temporal switch from M1 to M2 macrophages [13].

Cytotoxic CD8^+^ T cells participate in the immune response post-MI; however, they are primarily associated with clearing virally infected cells, can induce cardiomyocyte death in vitro [64] and have an important role in myocarditis. In terms of cardiac ischemia, genetic deletion of CD8^+^ T cells using CD8a*^tm1mak^*mice, decreased mortality after permanent coronary artery occlusion; however, the all-cause mortality in CD8a*^tm1mak^* mice was cardiac rupture [65]. Although survival was higher, MI in CD8a*^tm1mak^* mice resulted in defective efferocytosis, prolonged inflammation and poor scar formation, demonstrating the complex role of these cells in myocardial repair.

The recruitment of T cells to the heart indicates autoreactive immunity and antigen presentation of myocardial proteins by macrophages, dendritic cells or B cells to T cells. After MI, peptides released from cardiac tissue, such α-myosin heavy chain [66,67], promote the differentiation of naïve CD4^+^ T cells to assist in cardiac healing [67]. Of note, the autoreactive adaptive immune response after MI and their participation in wound healing is different from the chronic response seen in myocarditis, heart failure and aging. Sequencing of the T cell receptor (TCR) repertoire identified differences between patients with acute myocardial infarction or normal coronary arteries and, therefore, may have some prognostic value [68].

B cells are responsible for antibody production and, upon activation, are subdivided into plasma and memory B cells. Plasma cells actively produce antibodies while memory cells survive for years in a quiescent state but can quickly mount an immune response upon secondary exposure to an antigen. In the heart, B cells reside primarily in the cardiac vasculature and contribute to preservation of myocyte contractility and size [69]. A cardioprotective role for B cells within pericardial adipose tissues has also been described [70]. B cells may also have a maladaptive role in heart repair as they contribute to the acute immune response after MI by activating monocytes in the bone marrow [59]. The effect of B cell depletion therapy on patients with myocardial infarction using Rituximab, an antibody that targets CD20 on mature B cells and is used to treat autoimmune diseases and certain cancers, is currently in clinical trials (Rituximab in Patients with Acute ST-elevation Myocardial Infarction Study (RITA-MI) (NCT03072199)).

The classification of lymphoid cells has become increasingly complex and relies on appropriate cell surface markers. For example, a lymphoid cell which expresses markers of both T cells and NK cells (termed invariant NK T cells or iNKT cells) is required for the reparative phase after myocardial infarction [71]. A relatively new class of lymphoid cells, termed innate lymphoid cells (ILCs) has similar paracrine expression profiles to T helper cells but lack antigen-specific receptors [72]. Unlike other lymphoid cells, ILCs reside in tissues and undergo local activation and proliferation [73]. Elevated circulating levels of type 1 ILCs, which produce high levels of IFN-γ, were detected in patients experiencing an acute MI (within 12 h of symptom onset) [74]. Patient follow-up indicated that type 1 ILC abundance correlated with poor clinical outcomes. Type 2 ILCs are activated by cytokines such as IL-33 and produce IL-5 and IL-13 and have a diverse role in cardiovascular disease including: a pathogenic role in pericarditis via eosinophil activation, a beneficial role in atherosclerosis, and are implicated in cardiac remodeling post-MI [75,76,77]. Type 2 ILCs are a part of type 2 immunity which includes Th2 cells, M2 macrophages, eosinophils and mast cells. As a whole, the role of type 2 immunity on cardiac repair post-MI is unclear [78]. As the tools used to identify cell populations improve, our understanding of the role of lymphoid cells in cardiac disease is sure to grow and will undoubtedly lead to more questions.

## 3. Dysregulation of the Immune System during Aging

As the immune system has a fundamental role in heart repair post-MI, targeting the immune system is a practical approach to improving heart function after injury. A caveat to this approach, however, is the underlying influence of aging on cardiovascular and immune system function which could inadvertently impact the efficacy of therapies in differently aged populations. One hallmark symptom of aging is systemic, chronic inflammation, termed inflammaging [79] which becomes apparent in both the heart and bone marrow in the elderly. This type of inflammation is distinct from the acute inflammatory response seen after MI as it is a low-grade immune reaction and there is no pathogen or injured tissue. Franceschi and Campisi summarized several factors which contribute to inflammaging including: damaged macromolecules, microbial molecules (e.g., from the gut microbiota), mitochondrial dysfunction, cellular senescence, alterations of the coagulation system, immunosenescence and the complement pathway [80]. While aging impacts all cells, the rate of degeneration is cell- and tissue-specific [81,82]. The predominant cell population associated with inflammaging is hyperactivation of cells of the innate immune response, and a weakened adaptive immune system, with fewer naïve T cells and a smaller repertoire of T cell receptors [83]. IL-6, IL-1, TNF-α and IFN-γ are cytokines associated with cellular aging across tissues including the heart [84]. In fact, in an elderly cohort of over 2000 patients aged 70–79 years old, IL-6, C reactive protein (CRP), and TNF-α could be used as predictive markers of future cardiovascular events [85].

**Figure 2:**
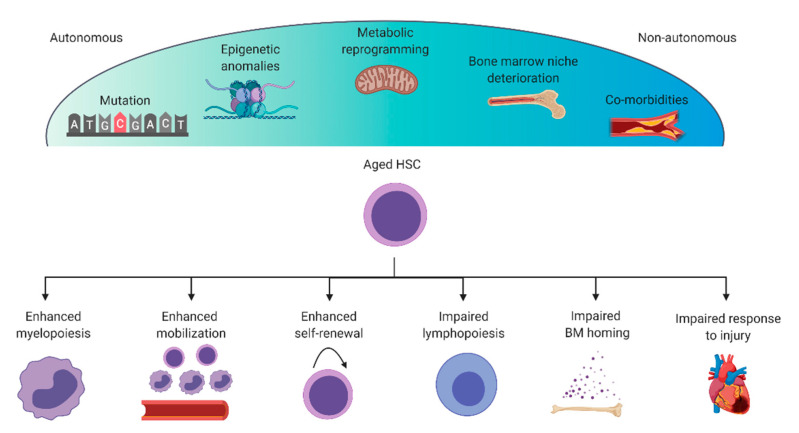
Mechanisms and consequences of immune cell aging. Cell intrinsic (autonomous) mechanisms of immune cell aging include mutations, epigenetic anomalies and metabolic reprogramming. Cell extrinsic factors that influence immune cell aging include deterioration of the bone marrow niche and co-morbidities, such as coronary artery disease or diabetes. With time, HSCs encounter various autonomous and non-autonomous drivers of aging that have downstream consequences on cell phenotype and systemic tissue function. Aged HSCs undergo enhanced myelopoiesis (myeloid skewing), mobilization to the circulation and self-renewal and show deficiencies in lymphopoiesis, BM homing and their ability to respond appropriately to tissue damage.

Why does the non-infarcted, aged heart develop chronic inflammation? Part of this etiology is due to cell fate decisions that arise within the bone marrow, when HSCs committed to the myeloid lineage preferentially expand over those committed to the lymphoid lineage [86]. From the lymphoid-myeloid perspective, this bias reduces overall lymphoid cell populations and amplifies myeloid cell populations which have become dysfunctional and promote cardiac inflammation [87,88,89]. Additionally, the lymphatic system, which has an important role in the response to myocardial infarction, deteriorates with age [90]. Many immunological disorders such as clonal hematopoiesis, myeloid skewing, and trained immunity are age-related phenomena which could amplify adverse cardiovascular events. While the intersection between cardiovascular health and immune cell activity is being re-examined, the underlying age-associated cellular changes have not been thoroughly studied. Below, we detail some mechanisms of aging that are putative targets for rejuvenation therapies (Figure 2).

### 3.1. Mutations and Epigenetic Anomalies

Age-related myelopoiesis, lymphopenia and anemia are driven in part by intrinsic (autonomous) cellular degeneration. Hallmarks of intrinsic drivers of HSC aging include increased LT-HSC proliferation, increased granulocyte-macrophage progenitor cells and fewer common lymphoid progenitor cells [91]. Autonomous cellular changes that drive these cell fate decisions include fundamental regulators of aging such as DNA damage [92,93], DNA methylation [94], telomerase dysfunction [95] and epigenetic anomalies [96]. These cellular changes underpin autonomous (intrinsic) HSC aging and can contribute to downstream consequences such as clonal hematopoiesis (which is dominant expansion of a mutated HSC or progenitor cell), lymphopenia, anemia, platelet hyperactivity and myeloid skewing.

Clonal hematopoiesis is an age-associated phenomenon which predisposes individuals to hematological cancer and cardiovascular disease [97,98]. To date, experimental manipulation of genes that promote clonal hematopoiesis have identified changes in monocytosis and macrophage accumulation [99]. Mutations in the proto-oncogene, Tet Methylcytosine Dioxygenase 2 (Tet2), an epigenetic regulator of DNA methylation leads to increased activity of myeloid cells, including polarization towards pro-inflammatory macrophages [99]. In addition to Tet2, mutations in DNA (cytosine-5)-methyltransferase 3A (DNMT3A), Additional Sex Combs-Like 1 (ASXL1) and Janus kinase 2 (JAK2) induce clonal hematopoiesis and cardiovascular disease [98]. Tet2, DNMT3A and ASXL1 are epigenetic regulators of gene expression underscoring the importance of epigenetic state in immune cell fate decisions. Bone marrow transplant from Tet2-deficient donor HSCs or myeloid progenitors increased IL-1β expression and exacerbated cardiac damage after permanent occlusion, highlighting the connection between clonal hematopoiesis and heart function [100].

At the top of the hematopoietic hierarchy are long-term reconstituting HSCs (LT-HSCs), thought to give rise to all blood cell lineages. LT-HSCs are predisposed to certain lineages, referred to as lymphoid-biased, myeloid-biased or megakaryocyte/platelet-biased HSCs. As HSCs age, their gene expression profile shifts to myeloid- and platelet-biased HSCs, and fewer lymphoid-biased genes are expressed [89,91]. The multipotent progenitor (MPP) cell is the point at which a common myeloid (CMP) or lymphoid progenitor (CLP) cell is formed. In adult mice, this number is already imbalanced with 1 MPP forming approximately 4 CMPs, but only 1 CLP is formed per 46 MPP [101]. Aging progressively reduces the CLP population, CLP proliferation and B cell development [91,102].

Microarray analysis of HSCs transcripts from 2-, 6-, 12-, and 21-month old mice identified broad upregulation in transcriptional programs associated with inflammation and epigenetic modifications, indicating altered functionality [96]. Genome-wide profiling of the histone modification, H3K4me3, and DNA methylation in 4- and 24-month old mouse HSCs demonstrated that the epigenetic state drives HSC cell fate decisions that favor self-renewal over differentiation [103]. Single cell approaches that incorporate genomics, transcriptomics and epigenomics have helped to elucidate the complex cell fate decisions that change with HSC aging. Single cell RNA-seq (scRNA-seq) profiling identified that old LT-HSCs spend less time in G1, indicating these cells progress faster through the cell cycle [104], in agreement with the established observation that HSC self-renewal increases with age. Lineage tracing of HSCs in combination with scRNA-seq shows that HSCs have a finite amount of myeloid, lymphoid or erythroid cell progenitors [105]. Interestingly, the authors also revealed that megakaryocytes develop from the most primitive HSCs, at the top of the hematopoietic hierarchy and not a more restricted progenitor. As megakaryocyte/platelet-biased HSCs increase with age [89], understanding this cell population is particularly important in age-associated disease.

In addition to cell fate decisions, aging also affects the mobilization and engraftment of HSCs and progenitor cells [106]. Although the migration of HSCs in response to CXCL12 is unchanged in 2- and 24-month old murine HSCs, Xing et al. demonstrated that it is the adhesion between stromal niche cells and HSCs that deteriorates [107]. In vivo, using a competitive reconstitution assay, an equal mixture of young and old HSCs demonstrated that aged HSCs have a reduced capacity to repopulate the bone marrow. Additionally, aged HSCs are not well retained in the bone marrow and are more susceptible to G-CSF mobilization. Part of this is explained by the age-related upregulation of Rho-GTPase Cdc42 activity, which has a role in proliferation, cell adhesion, microfilament organization and cell polarity. Genetic deletion of the Cdc42 inhibitor, Cdc42GAP, upregulated Cdc42 activity and, subsequently, reduced HSC adhesion to fibronectin [108]. Cdc42 activity is upregulated with aging and senescence in a number of tissues. Using the genetic knockout mouse of Cdc42GAP to upregulate Cdc42 activity resulted in premature aging of fat, bone, skin and muscle [109]. Cdc42 itself is polarized toward one end of LT-HSCs in young cells, but becomes apolar with age [110]. Interestingly, this localized Cdc42 activity is associated with polarity of nuclear histone 4 lysine 16 acetylation, in a term referred to as epipolarity. Inhibiting Cdc42 activity via pharmacological antagonists also shifts HSC polarity and rejuvenates histone 4 lysine 16 acetylation [110].

Further analysis showed that upstream regulation of Cdc42 activity is via Wnt5a signaling [111]. Both gain and loss of function approaches were used to demonstrate the potent role of Wnt5a in HSC aging. When young HSCs from 3-month-old mice are exposed to Wnt5a, classical molecular and functional symptoms of HSC aging develop including enhanced Cdc42 activity, myeloid skewing and reduced engraftment potential. In contrast, loss of Wnt5a in old HSCs, from 20–24-month-old mice, reversed these age-associated effects. Loss of Wnt5a function was assessed using both genetic deletion (haploinsufficiency) and short hairpin RNA (shRNA) mediated knockdown in LT-HSCs, which demonstrates Wnt5a promotes HSC aging in a cell autonomous fashion. Regulation of the HSC epigenome is complex. A comprehensive understanding of how epigenetic aging of HSCs and immune progenitor cell affects the immune system will be important in creating therapies to improve tissue repair.

### 3.2. Deterioration of the Bone Marrow Niche

Cell extrinsic, nonautonomous factors which contribute to HSC aging include remodeling of the bone marrow niche [112], including adrenergic drive, cellular composition, oxygen concentration, cytokine production and pre-exposure to tissue injury [113]. Niche cells include macrophages, osteoblasts, endothelial cells, megakaryocytes and mesenchymal stem cells (MSCs). Niche factors that regulate HSC activity include: CXCL12, Angiopoietin 1 (ANGPT-1), kit ligand (KITL aka Stem Cell Factor (SCF)), Thrombopoietin (TPO), TGF-β1 and Vascular cell adhesion protein 1 (VCAM-1) [114,115,116]. These cells and factors are also influenced by aging which can ultimately regulate HSC quiescence, proliferation and differentiation.

The signaling between bone marrow MSCs and HSCs have been one of the better characterized interactions. CXCL12, a retention factor that promotes HSC quiescence is produced by MSCs and megakaryocytes and acts on the CXCR4 receptor present on HSCs [114,117]. The systemic role of innervation on MSC-HSC communication has been particularly interesting. Using a Cgt knockout mouse to reduce neural input, Katayama et al. demonstrated that input from the sympathetic nervous system controls HSC quiescence by modulating the activity of MSCs and CXCL12 production [118]. Downregulation of neural input reduced HSC mobilization and lymphopoiesis. Further investigation of this pathway has shown that the signaling factor of the sympathetic nervous system, noradrenaline acts specifically on the β3-adrenergic receptor, expressed only on MSCs and not other niche cells and that CXCL12 production is regulated by circadian rhythms [119]. Megakaryocytes also contribute to HSC quiescence via CXCL4, CXCL12, TPO and TGF-β1 [115,116,117]. Regulating HSC bias by controlling the bone marrow niche cell activity has been linked to cardiovascular disease as, after MI, myelopoiesis is pushed into overdrive by noradrenaline signaling [16].

An additional consideration to HSC behavior is that the bone marrow is not homogenous. Spatial regions of the bone marrow are subdivided into the endosteal and vascular niches (including arterioles or sinusoids) which are near or far from the bone, respectively. The vascular BM niche is enriched in endothelial cells and Neural/glial antigen 2 (NG2^+^) periarteriolar pericytes and promotes HSC bias to the myeloid lineage while the endosteal niche promotes bias toward the lymphoid lineage [120,121,122]. The endosteal region was thought to be more hypoxic than the sinusoidal niche, however, using two-photon phosphorescence lifetime microscopy this was shown not to be the case [123]. With aging, the endosteal HSC niche shrinks and the perivascular, myeloid-biased HSC niche expands [112]. This is in part derived from adrenergic drive, as loss of the β3-adrenergic receptor in *Adrb3^−/−^* mice lead to expansion of myeloid-biased HSCs in the endosteal niche [112].

The interaction between HSCs and megakaryocytes in the BM niche also has a role in age-related HSC dysfunction which is regulated by noradrenergic signaling and macrophage inflammation. Megakaryocytes are normally found throughout the BM in both the perivascular and endosteal niches and promote HSC quiescence. With aging, the megakaryocyte-biased HSCs population increases [124]. Ho et al. demonstrated that expansion of the megakaryocytes in the BM niche was, in part, caused by age-related increase in noradrenergic signaling via the β2-adrenergic receptor (Ardb2), which upregulated expression of IL-6 in stromal niche cells [112]. The discrepancy between the concomitant increase in megakaryocytes [124,125] (which typically promote HSC quiescence) and HSCs with aging may be reconciled by changes in their physical interaction. The physical proximity between HSCs of the endosteal niche and megakaryocytes decreases with age, facilitating HSC activation [112]. This is regulated by an age-associated loss in β3-adrenergic receptor signaling, which could be corrected in progeroid mice using β3-adrenergic receptor agonist [112]. Thus, the interaction between aging, innervation and the BM niche cells on HSC function may be complex, but also offers the opportunity for therapeutic intervention.

### 3.3. Metabolic Adaptations

HSC and immune cell aging have been shown to be particularly sensitive to metabolic activity. Several metabolic therapies, such as caloric restriction, IGF-1 depletion, reactivation of autophagy or rapamycin treatment, have been shown to reduce myeloid bias, restore self-renewal and in some cases, increase lifespan [126,127,128].

Autophagy has a complex role in old LT-HSC subpopulations. In 24–28 month old mice, approximately one-third of LT-HSCs maintain autophagy via transcription factor, Forkhead Box O3A (Foxo3a) [128,129]. These cells are tolerant to metabolic stress and have shown long term engraftment potential [128,129]. The majority of old LT-HSCs have defects in autophagy. This is important as autophagy suppresses HSC metabolism which ensures HSC quiescence but also has downstream consequences on myeloid skewing [128]. Deletion of Autophagy Related 12 (*Atg12*) from young HSCs resulted in a premature aging phenotype and myeloid bias [128].

Modulation of the mitochondrial content is regulated by mitophagy (autophagy of mitochondria). Vannini et al. demonstrated that mitochondrial content could regulate HSC fate decisions [130]. Within the hypoxic environment of the bone marrow niche reside LT-HSCs which take on a unique metabolic profile that rely on anerobic glycolysis for energy, perfectly suited to their environment. More mature progenitor cells use oxidative phosphorylation [131]. Inhibition of mitochondrial activity prevented differentiation of HSC progenitor cells and instead converted them to a self-renewing state [130]. Using Nicotinamide adenine dinucleotide (NAD^+^)-boosting agent nicotinamide riboside (NR), Vannini et al. were able to improve HSC function and suggested this could be adapted to hematological and oncological diseases [132].

More mature or differentiated immune cells also undergo metabolic changes that control their behavior. M1 and M2 macrophages rely on glycolysis or oxidative phosphorylation during the early and late phases of repair post-MI, respectively [133]. The age-related changes in macrophage metabolism on pro-inflammatory phenotype is suspected, but more investigation is required [134]. The metabolic activity of T cells has been shown to have a leading role in systemic tissue inflammaging. Bharath et al. determined that the inflammaging of CD4^+^ T cells takes on a Th17-biased profile, with defects in autophagy and mitochondrial activity [135]. Using metformin, a drug used to treat type 2 diabetes, they were able to rejuvenate the metabolic profile of old T cells (collected from individuals approximately 60 years old) and subsequently reduce inflammation, as determined by reduced STAT3 activity and IL-17 levels. In young CD4^+^ T cells (collected from individuals approximately 30 years old), disrupting autophagy via siRNA targeting of autophagy-related protein 3 (*Atg3*) led to premature development of the Th17 phenotype.

### 3.4. Co-Morbidities and Sex

Recent studies have explored how environmental factors including diet, stress and previous cardiovascular events lead to epigenetic modifications and the downstream consequences on immune cell activity or differentiation [136]. When HSCs or progenitor cells are exposed to a certain stimulus, they undergo epigenetic and metabolic reprogramming and may retain those epigenetic marks and metabolic activities in a phenomenon referred to as trained immunity. An important feature of trained immunity is that it is unique to cells that participate in the innate immune response [137]. Upon secondary exposure, these innate immune cells are already primed to respond as they have formed epigenetic and metabolic memory from the previous encounter. This typically induces a secondary response that is stronger than the first [138]. In contrast to trained immunity is immune tolerance, in which after a second treatment (e.g., Lipopolysaccharides (LPS) treatment) the immune response is essentially paused and non-responsive to the second stimulus [139]. Ifrim et al. characterized the ligands which promote immune training or tolerance [140]. Cultured human monocytes treated with β-glucan or LPS to prime cells toward a trained or tolerant phenotype, respectively, induced stable epigenetic reprogramming that controlled macrophage phenotype upon monocyte differentiation [141]. Cremer et al. put the hypothesis of trained vs tolerant immunity to the test in the context of recurring ischemic heart disease [142]. To perform a recurrent MI in mice, they sequentially ligated the LCX and LAD arteries. After the second MI, the inflammatory response was muted in part because of bone marrow macrophages which retained memory of the previous injury [142]. The authors surmised that part of the failure of the second immune response is because of changes in signals coming from the heart and acting on the bone marrow niche. While this study did not directly link these effects to aging it is logical to infer that during aging, the risk of exposure to cardiovascular events increases. The accumulation of these events in aged cells could exacerbate negative cardiac effects.

Type 2 diabetes is a significant risk factor for cardiovascular disease that is associated with chronic inflammation (specifically myelopoiesis) and aging [143,144,145,146,147]. Part of this etiology is due to HSC fate decisions and bone marrow niche remodeling. In human type 2 diabetic bone marrow samples, the primitive CD34^+^ HSC population is decreased and shows higher rates of apoptosis compared to non-diabetics; however, more mature HSC populations were unaffected, indicating a potential reduction in self-renewal of the former. Mechanistically, this was attributed to reduced mir-155 expression and precocious FOXO3a activity in HSCs, which could by downregulated by exogenous expression of mir-155 [148]. Adipose tissue transplant from obese to lean mice showed that bone marrow myelopoiesis in obesity is driven by IL-1β produced by adipose tissue macrophages [145]. In contrast, HSCs in type 1 and 2 mouse models of diabetes are unable to mobilize HSCs in response to G-CSF as CXCL12 expression is abnormally maintained in MSC niche cells [149]. Albiero et al. showed that decreased HSC mobilization (mobilopathy) and myelopoiesis in diabetes are linked via p66Shc, which has dual roles as an adaptor for membrane proteins and also translocates to the mitochondria after phosphorylation where it contributes to oxidative stress [146]. By selectively deleting p66Shc from hematopoietic or nonhematopoietic cells (via bone marrow transplant of WT or p66Shc^−/−^ cells to p66Sch^−/−^ or WT mice, respectively) in diabetic mice they demonstrated that loss of p66Shc prevents myelopoiesis and mobilopathy. Mechanistically, the authors conclude p66Shc contributes to HSC maintenance by stimulating MSC production of CXCL12. It is unclear how p66Shc induces myelopoiesis though they observed that expansion of the megakaryocytic bone marrow niche cell population, which promotes myeloid-biased HSC development, was prevented in p66Shc knockout mice. Bone marrow niche endothelial cells reside in close proximity to HSCs in diabetic mice, modulating their function and myelopoiesis [147]. Interestingly, epidermal growth factor (EGF) signaling in endothelial cells modulates the typical immune cell response observed in diabetic mouse models, as endothelial-specific deletion of the EGF receptor (EGFR) prevented this effect. In this study, *Cxcl12* expression was reduced in endothelial cells; however, no change in *Cxcl12* expression was observed in MSCs. The two studies discussed here were observed under streptozotocin or diet-induced models of diabetes which could explain the conflicting reports of MSC activity and *Cxcl12* expression. Clearly, further investigation into the mechanism of bone marrow niche-mediated myelopoiesis or mobilopathy is required. A third type of bone marrow niche cell that has been linked to diabetes-induced inflammation are lymphoid cells which are more abundant in the bone marrow of type 2 diabetics. Abatacept, which inhibits T cell activation and shows clinical value in some type 1 diabetics [150] was able to reduce inflammation and improved cardiovascular function in a genetic model of diabetes [151]. Thus, in obesity and type 2 diabetes, the systemic environment is enriched in myeloid cells but unable to mobilize beneficial HSCs in response to stimuli, and the niche plays a vital role in these events [149].

Finally, sex has an important role on cardiac aging [152] and HSC behavior [153] wherein young pre-menopausal women are at a lower risk for cardiovascular events than age-matched male counterparts [154]. One factor at play in this context is estrogen which promotes HSC proliferation [155] and also regulates a number of terminally differentiated immune cells to reduce inflammation [156]. Interestingly, telomere length in leukocytes is preserved in women, regardless of menopause status, compared to men [157]. In the context of biological aging as assessed by a frailty index in aged rodents where middle aged and aged is defined as 12- and 30-months old, respectively [158], sex impacts cytokine levels in the blood such that IL-6, IL-9 and IFN-γ levels correlate with frailty in female mice, while IL-12 correlates with family in males [159]. These sex-related intersections are also found in other chronic inflammatory conditions including obesity. When placed on a high-fat diet, male mice are more prone to myelopoiesis in vivo and hematopoietic and progenitor cells are primed to response to LPS treatment in vivo [160]. In sum, the healthspan and genetic background varies on an individual basis and may influence HSC biological aging to impact future cardiovascular events.

## 4. Consequences of HSC Aging on Heart Repair and Prospective Therapies

Our understanding of immune cell function on cardiovascular events continues to advance. In 2004, it was reported that elevated leukocyte numbers may be used in risk factor assessment of coronary heart disease [161] but more recently, a detailed panel of 34 myeloid and lymphoid subpopulations from peripheral blood showed leukocyte quantity had no predictive value of future MI and correlate better with atherosclerosis [162]. A meta-analysis of 24 studies with 43,725 individuals, showed that shorter leukocyte telomere length, a bio-marker for aging, was associated with coronary heart disease [163]. As aging of HSCs and immune cells has a profound effect on their function, assessing these deficiencies in the context of the cardiovascular system is a logical next step and yet there are few studies that directly tie aging of the immune system to heart function and repair processes (Table 1). Logistically, targeting the immune response in the aged heart could be approached from different vantage points. That is, with aging, the heart develops an inflammaging profile, with enhanced leukocyte infiltration and cytokine production that contributes to local damage, yet aging is associated with a dampened but prolonged immune response after cardiac ischemia [164,165,166]. In other words, the aged heart has deficiencies in both cardiac homeostasis (e.g., chronic inflammation) and repair responses (e.g., ischemic injury). As the immune system has a pivotal role in cardiac function, controlling their behavior could lead to beneficial cardioprotective therapies.

### 4.1. Anti-Inflammatories

Early immunomodulatory cardiovascular therapeutics, such as steroids and NSAIDs, were used with the aim of limiting inflammation post-MI but instead increased infarct size during the early phase of repair, promoted scar thinning and caused an overall delay in healing [176,177,178]. In recent years, it is the understanding of the different aspects of the immune response and specific targeting of subpopulations to control repair, not necessarily targeting the overall immune response, that has garnered the most interest, and several prospective therapies have used this strategy in clinical trials.

In the context of aging, there are two sources of inflammation: from the source of injury (e.g., MI) and systemic inflammation (either caused by other co-morbidities or due to senescence). For example, cytokines IL-6 and TNF-α are upregulated in the heart post-MI, but also systemically with age [85,164]. Therefore, anti-inflammatory therapeutics must take both sources into account when considering the dose and duration of medication. Several clinical trials have established that neutralizing antibodies and small molecule inhibitors that target pro-inflammatory proteins are plausible avenues in limiting further tissue damage after MI. Examples of pilot and clinical trials include TNF-α antagonist (Etanercept) [179], IL-6 receptor antagonist (Tocilizumab) [180,181], IL-1 receptor antagonist (Anakinra), p38 MAPK inhibitor (Losmapimod) [182], methotrexate [183] and Lipoprotein-associated phospholipase A2 (Lp-PLA2) inhibitor (Darapladib) [184] all of which showed mild to moderate outcomes without significant clinical benefit.

The Canakinumab Antiinflammatory Thrombosis Outcome Study (CANTOS) repurposed the anti-arthritic drug Canakinumab, a neutralizing antibody of IL-1β, in stable patients with previous MI [185]. Canakinumab initially showed promise as the trial reported a reduction in subsequent cardiovascular events by 15% [185]. While this was a landmark result, there were some shortcomings. The clinical drawback to Canakinumab is an increased rate of fatal and nonfatal infection. Accompanied by the price of monthly administration of Canakinumab ($16,000 per injection), widespread buy-in waned [186]. In 2019, colchicine, a non-specific, affordable drug with anti-inflammatory properties and an established safety profile, was shown to have similar beneficial effects on cardiovascular end points (e.g., subsequent MI) as Canakinumab [187]. Importantly, while both patient cohorts had a previous MI, the Colchicine Cardiovascular Outcomes Trial (COLCOT) enrolled patients who had a previous MI within 30 days, while CANTOS enrolled stable patients. The repurposing of anti-inflammatories to cardiovascular disease remains an area of ongoing research with the potential to move quickly to the clinic if long-term safety can be achieved. It will also be important to understand the potency of these drugs in differently aged populations and how chronic inflammaging impacts their efficacy. Unfortunately, none of the agents under discussion specifically reduce cardiac inflammation or enhance cardiac regeneration. More directed therapies may improve clinical outcomes. In addition to the secretion of cytokines and chemokines, immune cells also secrete extracellular vesicles (EVs) which can, in turn, influence cardiac viability post-MI [188]. For example, EVs isolated from M1 activated macrophages induced neonatal rat cardiomyocyte death in-vitro in an NF-κB-dependent manner, suggesting that the adverse effects of M1 cells on cardiac viability may in-part be mediated through EVs [189]. As aging influences EV cargo, including microRNA that regulates inflammatory pathways [190], their use as biomarkers, targets or treatments in cardiovascular aging may offer more robust opportunities to improve health.

### 4.2. Regulation of Immune Cell Quantity and Diversity by Directing Cell Fate Decisions

In the context of aging, it is also important to consider whether the intensity and/or duration of the cellular infiltrate post-MI can be controlled to mount a more appropriate response. Aging affects both the macrophage population and function: cardiac resident CCR2^−^ macrophages, which support heart function via angiogenesis and perhaps cardiomyocyte proliferation (in the adult) [43], are replaced by CCR2^+^ macrophages, from blood-derived monocytes [17,52,191]; resident cardiac macrophages become biased toward a fibrotic, senescent phenotype [169]; and M1 macrophages increase while M2 macrophages decrease [192]. Persistent myelopoiesis has significant clinical impact as it is associated with ischemic heart disease and stroke [16], chronic stress [193] and aging [194]. Therefore, controlling cell recruitment, activity and immune cell fate decisions are of great interest.

Based on the M1/M2 macrophage dichotomy there have been numerous attempts to alter the timing and/or duration of monocyte or macrophage activity to preserve cardiac function. For example, genetic deletion of MMP9 reverses the age-related shift in the M1/M2 ratio in the heart [192]. Myocardial infarction in MMP9 knockout mice form 11 to 36 months of age showed improved survival and reduced left ventricular dilatation due to upregulation of M2 macrophage activity [175]. More precise targeting of macrophage polarization is also possible. Nanoparticle delivery of siRNA targeting the transcription factor, IRF5, which regulates macrophages polarization during the inflammatory phase of cardiac repair after MI, shifted the macrophages profile away from M1 and improved infarct healing [195]. Nanoparticle delivery of pharmacological agents has also been administered post-MI. Delivery of a chemical inhibitor of Toll-like receptor 4 (TLR4) in an I/R model of MI decreased inflammation and infarct size and improved overall heart function [196].

Given the ongoing activity of HSCs and their progeny, targeting aged immune cell function at the epigenetic level is an intriguing possibility. Transcriptional analysis of circulating leukocytes showed that patients with MI had reduced expression of the long noncoding RNA (lncRNA), nuclear enriched abundant transcript 1 (NEAT1) [197]. Using a knockout model, *NEAT1^−/−^* mice developed an exaggerated inflammatory response, though the mice were not challenged by coronary occlusion. Targeting histone post-translational modifications to influence cell phenotype is also possible. Using primary human LPS-stimulated macrophages, Kruidenier et al. demonstrated that inhibition of H3K27 demethylases of the KDM6 subfamily could block inflammation [198]. Inhibition of the KDM6 subfamily preserved the repressive chromatin modification, H3K27me3, on pro-inflammatory genes such as TNF-α. They extended their experiments to macrophages from patients with rheumatoid arthritis and found that TNF-α was also reduced by this approach. Chromatin modifying enzymes also play a pivotal role in M1 and M2 polarization. LPS-activated M1 macrophages express H3K4-specific methyltransferase mixed lineage leukemia (MLL) and H3K27 demethylase KDM6B while M2 macrophages express DNA methyltransferases (DNMTs) and histone deacetylases (HDACs) which promote and repress gene expression, respectively [199].

Cell therapies used to treat cardiovascular disease include intramyocardial or intracoronary delivery of myogenic cells (e.g., MSCs or cardiomyocytes derived from induced pluripotent stem cells), bone marrow mononuclear cells and endothelial progenitor cells. Unfortunately, clinical trials demonstrated that the efficacy of bone-marrow-derived cell therapy is low [200]. Importantly, however, those that do engraft, function in a paracrine manner to stimulate endogenous cell behavior. Recent evidence suggests that the benefits observed from cell therapy is primarily caused by the indirect recruitment of CCR2^+^ and CCR2^+^CX_3_CR1^+^ macrophages to the ischemic heart [201]. Vagnozzi et al. (2020) demonstrated that the innate immune response is activated by delivery of bone marrow mononuclear cells or cellular debris from dead bone marrow mononuclear cells results in similar functional improvements after I/R [201]. The use of zymosan, a chemical inducer of the innate immune response also improved heart repair. To prove that delivery of stem cells activates the immune response to improve heart function, the use of cyclosporine A, an immunosuppressive or chlodronate (macrophage depletion) was applied and the beneficial effects were lost. Functional improvements were due to reduced fibrosis. They concluded that activation of the immune response is the mechanism behind the functional benefits observed with cell therapy.

Notably, while aging reduces global lymphoid populations, sub-populations of lymphoid cells differentially expand and in the murine heart, CD4^+^ T cells increase with age [168]. In humans, changes in telomere length and the epigenetic landscape of CD8^+^ T cells has been associated with aging and coronary heart disease [202,203,204]. Using a 5 and 12 year follow-up, Lin et al. monitored telomere length and telomerase activity in subjects aged 20–90 years old and found that the lymphocyte population is particularly sensitive to telomere attrition [202]. Interesting, CD8+ T cell telomere shortening was also identified as a risk factor for coronary artery disease, independent of aging [203]. To assess how chromatin accessibility changes with immune cell aging, the assay for transposase-accessible chromatin with sequencing (ATAC-seq) was performed in combination with RNA-seq on whole peripheral blood mononuclear cells and individual immune cell populations from young (22–40 year) or old (>65 years old) subjects [204]. In increasing order, monocytes and naïve B cells showed no change, CD4^+^ T cells showed minimal change and CD8^+^ T cells showed dramatic changes in chromatin landscape with age. What consequences these observations have on improving cardiovascular health are yet to be investigated.

### 4.3. Bone Marrow Transplant and Niche Remodeling

The foundation for bone marrow transplant was established in the 1950s and is primarily used to treat bone marrow oncological diseases. Based on the systemic influence of bone marrow HSCs on tissue function, in theory, replacing old bone marrow progenitor cells with young cells could restore tissue function. This procedure typically involves radiation and allogeneic bone marrow transplant which is difficult in humans without malignancies. Partial bone marrow replacement with or without low-dose radiation and induction therapy have been employed for benign disease [205,206,207]. However, studies in mice show the benefits of bone marrow replacement in models of aging or chronic disease. Heterochronic (young to old) bone marrow transplant in 20–22 month old mice improves angiogenesis via upregulation of *Cxcl12*, *Vegf* and the inflammatory response post-MI [172,174,208]. Young bone marrow cell transplantation can also improve cognitive function and muscle repair in aged mice, implying a greater scope for bone marrow cell aging on systemic tissue aging [209,210,211]. Of note, cognitive improvements in old mice with young bone marrow is caused by changes in both neurons and microglia, the immune cell population in the brain [209,212]. Targeting circulating immunological factors which correlate with age and negatively influence cognition, such as β-2-microglobulin or Ccl11 [213,214] have been proposed to be putative targets that may limit age-associated disease. Targeting bone-marrow-derived molecules via inhibitors such as neutralizing antibodies may be a more feasible approach as it circumvents bone marrow transplant; however, it implies that targeting one or a few molecules is sufficient to limit the negative effects of aging.

Based on these systemic findings, the effect of aged hematopoietic and immune progenitor cells on cardiovascular health is a field ripe for investigation. It is the delivery method and subsequent stable engraftment of these cells to the bone marrow that is the limiting factor in moving this type of cell therapy from the lab to the clinic. To circumvent the toxic effect of radiation, other models of HSC replacement therapy have been proposed including nonmyeloablative transplantation, anti-cKit and anti-CD47 antibody-mediated HSC depletion or mobilization based-transplant [215,216,217]. In nonmyeloablative transplantation, the therapy relies on our understanding that the signals which retain HSCs to the niche are defective with age [107]. In theory, this provides an opportunity for transplanted young HSCs to home to the aged BM niche. In 2005, Kamminga et al. demonstrated that indeed young HSCs could take up residence in the BM of an aged mouse [218]. While the strategy can extend rodent lifespan, the efficacy is still low, potentially because of the age-related expansion of bone marrow MSCs [124]. However, using this approach, Kovina et al. showed that transplantation of whole BM, not just purified HSCs, resulted in 28% chimerism 6 months post-transplant [215]. They attributed this high rate of chimerism to the age of the recipient (B10-GFP mice, 15 months old). Mobilization-based therapies, such as combination G-CSF and AMD3100, increases the rate of HSC deployment and temporarily empties the BM but preserves the niche. Using this method, heterochronic transplant of young HSCs prolonged lifespan, even with a lower reconstitution rate than traditional myeloablative therapies. Arguably, even with a low rate of reconstitution, incorporation of young LT-HSCs could have significant beneficial outcomes. Mixed chimerism even at a low rate may be sufficient to permit cardiac restoration in an aged individual after coronary occlusion. Bone marrow transplantation with young cells in aged hosts has distinct advantages over cell transplantation because stable engraftment and persistent effects can be demonstrated with bone marrow transplants [172,173]. In addition, bone marrow transplantation leads to stable engraftment of young bone marrow cells in the heart. This treatment has great promise if partial chimerism is sufficient to alter the response to cardiac injury.

In addition to cell replacement therapy, it may be possible to rejuvenate resident HSCs or other bone marrow resident cells using with pharmacological agents. This may be approached from several avenues which have shown promise in pre-clinical anti-aging studies including targeting adrenergic signaling, epigenetic reprogramming or redirecting metabolic activity [110,126,219,220]. For example, macrophages are also present as resident cells in the bone marrow and with aging become are defective in efferocytosis but are also hyperactivated, releasing high levels of IL-1β [124]. Increased IL-1β induces megakaryocyte-bias in HSCs but this effect could be reduced ex vivo using anakinra, an IL-1R1 antagonist. As any downstream impacts of bone marrow therapeutics on cardiovascular function have not been adequately investigated, this field could lead to some interesting developments in lifespan and healthspan.

### 4.4. Senolytics

Senescence is a cellular state commonly characterized by cell cycle inhibition, increased cell secretory activity, and changes in cell morphology [221]. With increasing age, there is an accumulation of these dysfunctional cells within tissues and this accumulation is associated with organ dysfunction in multiple organ systems. The deleterious effect of senescent cells is primarily attributed to increased secretion of pro-inflammatory cytokines, chemokines, growth factors, extracellular vesicles, and proteolytic enzymes by these cells which, in turn, disrupts tissue microenvironments and promotes cellular dysfunction [190,221,222]. This unique secretory profile is termed the senescent-associated secretory profile (SASP). Cellular senescence has been shown to have indirect and direct effects on immune cell populations involved in infarct healing post-MI. For example, senescent cells promote the expansion of immature myeloid cells which, in turn, further alter resident cell function; this effect was shown to occur in an IL-6-dependent manner [223]. Immune cell subsets also acquire senescent cell states in a process termed immuno-senescence. Both innate and adaptive immune cells have been shown to exhibit some aspect of cellular senescence, such as increased expression of cell cycle checkpoint inhibitors (e.g., p16; CDKN2A), increased pro-inflammatory cytokine secretion, and a loss of cell function [224]. With aging, T-cells exhibit a decline in replicative potential and subsets can acquire a pro-inflammatory phenotype [225]. Collectively, cellular senescence has both indirect and direct effects on immune cells which together contribute to immune cell dysfunction in aged individuals. Recently, new therapies targeting senescent cells have emerged as a new way to improve immune cell function in aged individuals.

In a series of landmark studies, Van Deursen and colleagues demonstrated that systemic clearance of p16^+^ cells in transgenic mice carrying an INK-ATTAC construct, which allows for selective elimination of p16^+^ cells upon AP20187 administration, rejuvenated multiple organ systems of mice with an accelerated aging phenotype, as well as naturally old mice [226,227]. These studies demonstrated that tissue dysfunction which occurs with aging could be partially restored or rejuvenated following removal of senescent cells. This subsequently promoted the creation of a new drug class termed “Senolytics” which describes compounds that promote the removal or clearance of senescent cells throughout the body. Using high throughput and bioinformatics approaches candidate compounds have been identified which promote the death of or suppress the senescent phenotype of these cells. For example, inhibitors of the Bcl-2 protein family such as ABT263 and ABT737 have been shown to reduce p16^+^ cells in vivo by inhibiting the pro-survival pathways upregulated in senescent cells thereby promoting cell death [228,229]. Another pharmacological approach which has shown promise is a dasatinib and quercetin (D+Q) combination therapy [230]. D+Q treatment has been shown to improve vascular function, exercise capacity, and increase the lifespan of female mice [230,231,232]. A number of other compounds have also been identified with senolytic activity, as well as non-pharmacological approaches [233,234,235,236].

Although the number of studies is limited, pre-clinical mouse studies have demonstrated that senolytics can improve baseline cardiac cell function and cardiac repair in aged animals. In the uninjured heart, both ABT263 [237] and D+Q [238] therapies were shown to increase cardiomyocyte proliferation and reduce fibrosis in old mice (>20 months old). Although the mechanisms responsible for these effects remain unknown, removal of senescent cell paracrine factors in both studies was suggested to play a role. Removal of senescent cells also improves cardiac repair; administration of ABT-263 to aged mice improves survival and cardiac function post-MI compared to vehicle-treated mice [171]. Although the underlying mechanisms were not investigated, another study found that ABT263 can reduce myeloid skewing and rejuvenate bone marrow HSC function [228]. This suggests that the removal of senescent cells can improve cardiac repair post-MI through rejuvenation of immune responses. Consistent with this notion fisetin, a flavonoid similar to quercetin, reduces pro-inflammatory cytokine and p16 expression in peripheral CD3^+^ T-cells [236]. Interestingly, phase 1 clinical trials suggest that senolytic therapies may be effective in humans. D+Q therapy reduced the expression of SASP factors in an open label phase 1 clinical trial involving patients with diabetic kidney disease and improved markers of physical function in patients with idiopathic pulmonary fibrosis [239,240].

One important concept that has yet to be investigated is the timing of senolytic treatment post-MI. While initial studies have demonstrated that rejuvenation prior to MI improves repair in aged animals, no post-MI treatment studies have been performed. Following infarction fibroblasts become senescent in attempt to limit fibrotic responses [241]. Thus, removal of senescent cells may have different effects on repair processes activated and more studies are needed to determine what effect post-MI treatment can have on repair. Although much remains unknown about how different cell populations are affected, the safety profile, and duration of benefits of senolytic therapy, these studies support further investigation into the use of senolytics for rejuvenating inflammatory responses to improve cardiac repair in aged individuals.

## 5. Conclusions and Perspectives

**Figure 3:**
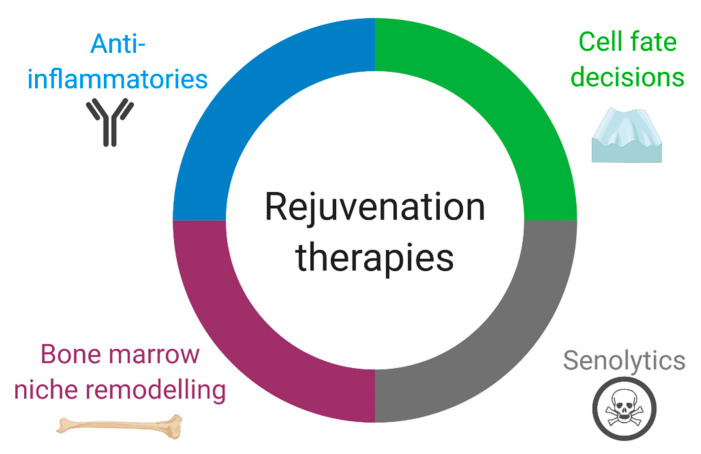
Putative rejuvenation therapies to reverse HSC and immune cell aging.

The heart relies on the activity of bone-marrow-derived cells for homeostasis and repair. With aging, systemic changes in the bone marrow and cardiac-resident immune cells can lead to maladaptive cardiac remodeling. Rejuvenation of the aged immune system via diverse immunotherapies have shown great promise in pre-clinical research but most have not been directly studied in the context of cardiac function. As these therapies advance, the role of aging on tissues and immune cells should be taken into consideration. Strategies that reverse immune cell aging in pre-clinical and early clinical trials include bone marrow replacement, bone marrow niche remodeling, regulation of immune cell diversity, anti-inflammatories and senolytics (Figure 3). As we understand how immune cell aging affects cardiovascular health, the translation of these studies into the clinic may become possible.

## Figures and Tables

**Table 1:** A brief summary of the effect of aging on cardiac immune cell infiltrate or repair processes.

Method	Injury	Outcome	Reference
8 wk., 18- and 30-mo. (WT)	N/A	Cardiac macrophages and neutrophils increase with age	[167]
2–3 vs. 12–15 mo. (WT)	N/A	Higher T cell activity in the heart draining lymph nodes	[168]
Cardiac macrophages from 4-, 8-, or 30-wk. (WT)	N/A	Functional and transcriptional profiling indicate a senescent, fibrotic phenotype forms	[169]
Heterochronic parabiosis	N/A	Reduced age-related cardiac hypertrophy	[170]
2–3 and >24 mo. (WT)	I/R	Impaired inflammation and healing; decreased cardiac function	[166]
Senolytic (ABT-263) administration; 23 mo. (WT)	P	Improved survival and cardiac function	[171]
Heterochronic BMT (2–3 vs. 20–22 mo.)	P	Enhanced angiogenesis, scar thickness and overall cardiac function	[172,173,174]
Competitive BMT using Tet2^−/−^ cells	P	Upregulated IL-1β expression; Increased fibrosis; Decreased heart function	[100]
MMP9 KO mice; 11–36 mo.	P	Enhanced M2 macrophage activity; improved survival; reduced left ventricular dilatation.	[175]

N/A = Not Applicable (no injury was performed); P = permanent coronary artery occlusion; I/R = ischemia/reperfusion; BMT = Bone Marrow Transplant; KO = Knockout; WT = Wild type; Mo. = month; Wk = week.
